# Applications of Resting-State fNIRS in the Developing Brain: A Review From the Connectome Perspective

**DOI:** 10.3389/fnins.2020.00476

**Published:** 2020-06-03

**Authors:** Zhishan Hu, Guangfang Liu, Qi Dong, Haijing Niu

**Affiliations:** State Key Laboratory of Cognitive Neuroscience and Learning, Beijing Normal University, Beijing, China

**Keywords:** connectome, graph theory, functional connectivity, development, NIRS

## Abstract

Early brain development from infancy through childhood is closely related to the development of cognition and behavior in later life. Human brain connectome is a novel framework for describing topological organization of the developing brain. Resting-state functional near-infrared spectroscopy (fNIRS), with a natural scanning environment, low cost, and high portability, is considered as an emerging imaging technique and has shown valuable potential in exploring brain network architecture and its changes during the development. Here, we review the recent advances involving typical and atypical development of the brain connectome from neonates to children using resting-state fNIRS imaging. This review highlights that the combination of brain connectome and resting-state fNIRS imaging offers a promising framework for understanding human brain development.

## Introduction

The human brain undergoes rapid development during the first few years (Gao et al., 2015a; Xiao et al., 2016). Non-invasive techniques such as functional MRI (fMRI) have shed light on neural development across the life span (Bunge et al., 2002; Zuo et al., 2017; Mohammadi-Nejad et al., 2018; Koen and Rugg, 2019). Combined with the newly developed connectomics framework, it characterizes the changes in neural network architecture during typical development or network dysfunctions underlying neurological disorders (Cao et al., 2017b). For example, small-world structure with efficient information segregation and integration is enhanced during development (Supekar et al., 2009; Wu et al., 2013; Cao et al., 2014). Furthermore, brain networks exhibit region-specific development, in which the primary sensorimotor and auditory regions develop first, followed by the visual, attention, default mode, and executive control networks in sequence (Gao et al., 2015a,b; Cao et al., 2017a).

Despite the remarkable fMRI findings, the operation cost, and extreme body confinement during the scan limit its use in neural developmental studies. Hence, the functional near-infrared spectroscopy (fNIRS) has been increasingly used for neuroimaging research in children. Compared with fMRI, fNIRS can be operated in a more economic, comfortable, quiet, and portable way, which is highly suitable for children participants (Cao et al., 2015). Moreover, fNIRS measures concentration changes in both oxyhemoglobin (HbO) and deoxyhemoglobin (HbR) with a much higher time resolution than fMRI, which provides more information about the neurovascular changes in the developing brain.

Resting-state fNIRS measures spontaneous hemodynamic fluctuations in the cortex and does not require explicit cognitive processes and task performance. This technique is feasible (Niu et al., 2012), reliable (Niu et al., 2013; Wang et al., 2019), and reproducible (Niu et al., 2011) in characterizing brain connectivity network at a spontaneous state. In the last decade, resting-state fNIRS has been increasingly utilized to delineate typical and atypical development in cortical functional connectivity and network topological characteristics, from neonates to adolescents.

The current review aims to summarize the current advances in the application of resting-state fNIRS in neural developmental research and is organized into three main sections. First, fundamentals of the fNIRS brain connectome is introduced. Second, typical and atypical development of fNIRS brain connectome was summarized. Specifically, we demonstrate the development of functional connectivity, network topologies, and network asymmetry in typical development children, as well as the brain connectome in preterm infants and children with neurological disorders. Finally, we discuss the remaining challenges and highlight future directions in this field.

## Literature Selection

**Figure 1:**
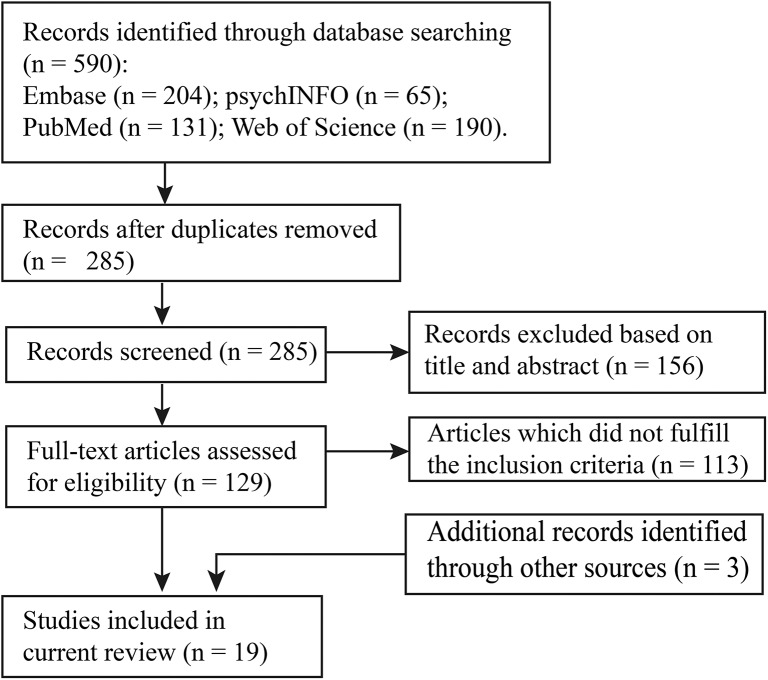
Flowchart of study selection.

In this review, we conducted a literature search on *PubMed, psychINFO, Embase*, and *Web of Science* databases using the following search items: [(NIRS OR fNIRS) OR optical] AND [(resting-state AND functional connectivity) OR (resting-state AND functional networks)]. We selected the studies that (a) used resting-state functional near-infrared spectroscopy; (b) recruited participants under 18 years old; and (c) characterized brain connectome patterns. With this procedure, 16 studies were identified for the systematic review. In addition, three articles were retrieved by manually checking the references in these studies and other relevant reviews. Details of the study selection are provided in Figure 1, and the included studies are summarized in Table 1.

**Table 1:** Overview of the included studies.

	**Study**	**Subjects**	**Age**	**N**	**Brain regions**	**State**	**Duration** **(min)**	**Instrument**	**Sources/** **detectors**	**#CH**	**Metrics**
Typical	Ferradal et al. (2016)	Infant	<2 D	9	Temporal, occipital, inferior parietal	Sleep	20 – 60	HD-DOT	32 S/34 D	NR	FC
	Homae et al. (2010)	Neonate Infant Infant	2 – 11 D 102 – 123 D 180 – 206 D	15 21 16	Frontal, temporal, parietal, occipital	Sleep	3	ETG-7000	30 S/30 D	94	FC
	Taga et al. (2018)	Infant Infant Infant Infant	71 – 100 D 101 – 130 D 131 – 170 D 291 – 330 D	25 28 24 14	Frontal, temporal, occipital	Sleep	10	ETG-100	8 S/64 D	48	FC
	Homae et al. (2011)	Infant	104 – 123 D	21	Frontal, temporal, occipital, temporoparietal	Sleep	3	ETG-7000	30 S/30 D	94	FC
	Funane et al. (2014)	Infant	113 D	9	Temporal	Sleep	2.5	ETG-100	2 S/8 D	8	FC
	Blanco et al. (2018)	Infant	124.6 ± 3.76 D	24	Frontal, temporal, parietal, occipital	Sleep	11 – 21	NIRScout	14 S/19 D	46	FC
	Bulgarelli et al. (2019)	Toddler	553.11 ± 12.17 D	43	Frontal, temporoparietal	Fixation	1.5	UCL-NIRS	12 S/12 D 16 S/16 D	30 44	FC
	Gallagher et al. (2016)	Early childhood Late childhood Adolescence	3 – 6 Y 7 – 10 Y 11 – 16 Y	6 8 11	Frontal, temporal	Fixation	5	Imagent	8 S/64 D	NR	FC
	Wang et al. (2017)	Children	6.9 – 8.21 Y	53	Frontal, temporal, parietal, occipital	Awake, eyes closed	11	CW6	12 S/24 D	46	Topology
	Cai et al. (2019)	Children	7.0 – 8.9 Y	30	Frontal, temporal, parietal, occipital	Awake, eyes closed	11	CW6	12 S/24 D	46	Topology
	Cai et al. (2018)	Children Adolescent	7.0–8.9 Y 11.0–12.9 Y	30 30	Frontal, temporal, parietal, occipital	Awake, eyes closed	11	CW6	12 S/24 D	46	Topology
	Koen and Rugg (2019)	Children	9.0 ± 1.5 Y	12	Frontal, temporal	Awake, eyes closed	8	FOIRE-3000	16 S/16 D	44	FC
Atypical	White et al. (2012)	Preterm Preterm (stroke)	30 – 39 W (GA) 40 W (GA)	4 1	Occipital	Sleep	10 – 20	HD-DOT	18 S/16 D	108	FC
	Imai et al. (2014)	Preterm Down's	28.9 ± 3.0 W (GA) 38.0 ± 1.6 W (GA)	15 5	Frontal, temporal, occipital	Sleep	6	ETG-100	6 S/6 D	10	FC
	Fuchino et al. (2013)	Preterm	23.1 – 36.7 (GA)	25	Frontal, temporal, parietal, occipital	Sleep	3 – 6.5	ETG-7000	30 S/30 D	94	FC
	Li and Yu (2018)	ASD	2.0 – 8.9 Y	46	Frontal, temporal, occipital	Fixation	10	LABNIRS	16 S/16 D	44	Topology
	Zhu et al. (2014)	ASD	9.0 ± 1.3 Y	10	Frontal, temporal	Awake, eyes closed	8	FOIRE-3000	16 S/16 D	44	FC
	Li et al. (2016)	ASD	9.3 ± 1.4 Y	25	Temporal	Awake, eyes closed	8	FOIRE-3000	16 S/16 D	44	FC
	Cao et al. (2015)	Cerebral palsy	10.2 ± 2.1 Y	6	Sensorimotor	Awake, eyes open	3	CW6	16 S/32 D	84	FC

For typical and atypical developmental trajectories, the studies are listed in ascending order of the mean age of the youngest group, respectively. For preterm infants, the gestational age (GA) is reported.

*ASD, autism spectrum disorder; D, days; Y, years; N, sample size; S, sources; D, detectors; #CH, number of channels; FC, functional connectivity*.

## Fundamentals of the fNIRS Brain Connectome

### fNIRS Imaging and Brain Connectome Construction

**Figure 2:**
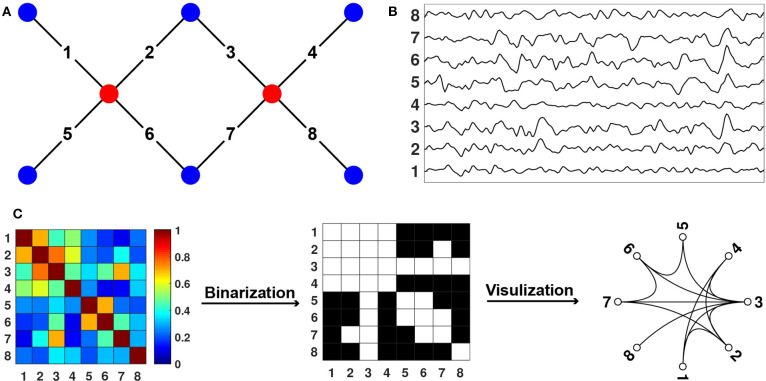
Diagram for fNIRS Data Collection and Processing. **(A)** Sources, detectors, and channels. The red dots represent the sources, and the blue dots represent the detectors. The solid black lines linking the red and blue dots represent the channels. **(B)** Changes in hemoglobin concentration signals from each channel. **(C)** Connectome calculation and construction.

fNIRS measures hemodynamic changes in the cerebral cortex induced by neural activity. In specific, NIR light emits from light source probes that can penetrate through the scalp and skull and arrive at cerebral cortex tissue. The reflected lights from the cortex tissue can be received and quantified by the detectors placed on the surface of the scalp (Figure 2A, Niu et al., 2010). Based on the Beer-Lambert law, the optical signals can be transformed into the concentration changes of the oxygenated hemoglobin (HbO) and deoxygenated hemoglobin (HbR) (Figure 2B, Delpy et al., 1988). To measure the hemodynamics emanating from spontaneous neural activity, the hemoglobin concentration signal is filtered with a bandpass filter which usually ranges from 0.009 to 0.08 Hz (Niu et al., 2012). Subsequently, spontaneous functional connectivity is calculated with Pearson correlation between the time series of every pair of nodes (i.e., measurement channels), which results in a correlation matrix. The correlation matrix is then thresholded into a binary matrix that describes the topological organization of the functional networks (Figure 2C). Of note, there are other approaches used to get brain connectivity networks, such as clustering (Homae et al., 2010; Blanco et al., 2018) and independent component analysis (White et al., 2012; Ferradal et al., 2016; ICA).

### Graph-Theoretical Topology Analysis of the fNIRS Brain Connectome

Graph-theoretical topology analysis is an ideal tool for characterizing network structure at global and regional levels (Cao et al., 2017b). At the global level, it has been well-established that human brain networks exhibit small-world topology (Watts and Strogatz, 1998), which are both locally and globally efficient, reflecting optimized information segregation and integration. At the regional level, regional nodal properties are usually evaluated by nodal degree and nodal efficiency (Niu and He, 2014; Cao et al., 2017b; Cai et al., 2018). For more details about graph metrics, see (Niu et al., 2012; Niu and He, 2014). For the analysis of fNIRS brain network, please see the freely downloaded software, FC-NIRS (http://www.nitrc.org/projects/fcnirs; Xu et al., 2015).

### fNIRS Brain Connectome Asymmetry

Hemispheric asymmetry is an important characteristic of the human brain. Recent studies have showed the topological asymmetry of structural or functional hemispheric networks in the human brain. The construction and topological analysis of the hemispheric network are conducted within each brain hemisphere, using the same methods applied to the whole-brain network. Usually, a brain asymmetry index (*AI*) is utilized to quantify the asymmetry in topological properties between two brain hemispheres (Cai et al., 2019). Specifically, the *AI* was defined using the following formula:

(1) $$\begin{array}{l} AI=\frac{ML-MR}{ML+MR} \end{array}$$

where the *ML* denotes the averaged measures in the left hemisphere and *MR* denotes the averaged measures in the right hemisphere. Therefore, the *AI* changes from −1 to +1, with a positive value denoting a leftward asymmetry and a negative value denoting a rightward asymmetry.

## Performance Evaluation of the fNIRS Brain Connectome

The performance of the fNIRS brain connectome has been evaluated by comparing its stability between different scanning time and by comparing its spatial patterns with other neuroimaging modality.

**Figure 3:**
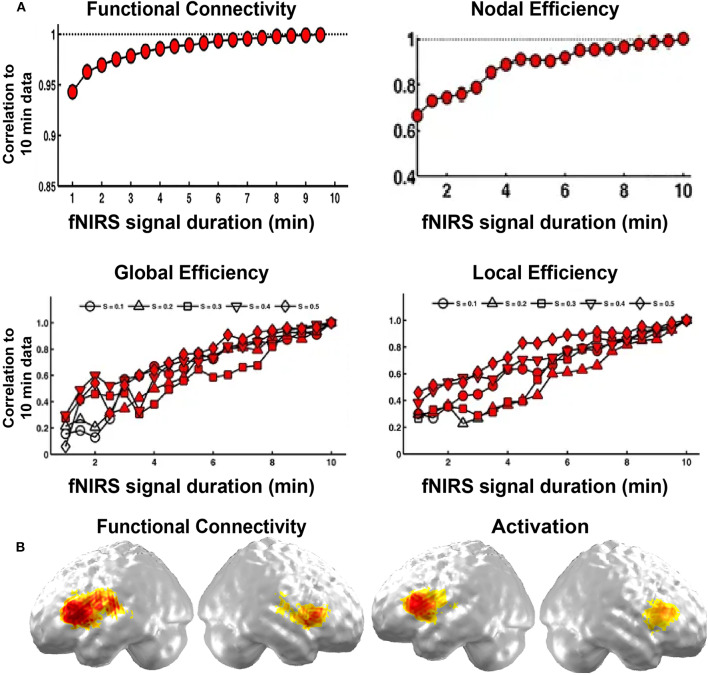
Validity and reliability of the brain connectome. **(A)** Correlations in network metrics, including functional connectivity, nodal efficiency, global efficiency, and local efficiency, between different short recording durations and 10 min of recording duration. The red-filled shapes represent significant correlations. **(B)** Functional connectivity and activation. **(A,B)** are adapted from Wang et al. (2017) and Gallagher et al. (2016), respectively, under a Creative Commons Attribution 4.0 International License (CC BY 4.0, http://creativecommons.org/licenses/by/4.0/).

In specific, the length of data acquisition time may affect the results in resting state imaging. Wang et al. (2017) explored its effect on the stability of fNIRS brain network on children participants. They found that FC was stable after 1 min fNIRS imaging duration, and both accurate and stable after 7 min duration. For network metrics, the minimum scanning duration of 2.5 min could achieve both accurate and stable performance (Figure 3B). This research provides direct evidence for the choice of the resting-state fNIRS imaging time in children in brain FC and network topology study.

In addition, the fNIRS brain connectome is often validated by fMRI imaging, ICA results, and task-evoked activation. For example, Ferradal et al. (2016) showed that spatial patterns in the functional connectivity in visual, middle temporal, and auditory areas were consistent with those obtained from resting-state fMRI imaging data. They also found that functional connectivity in these areas exhibited similar spatial patterns to those identified by ICA, and the networks were sensitive to neuronal injury identified by structure MRI (Ferradal et al., 2016). In addition, the functional connectivity map in the language network was also consistent with the task-evoked activation map (Figure 3A; Gallagher et al., 2016). These results validate the fNIRS brain connectome as a promising tool to characterize the brain network structure.

## Typical Development of the Brain Connectome

The typical development of fNIRS-derived brain connectome is summarized in terms of functional connectivity, topological network characteristics, and the asymmetry between hemispheric brain networks.

### Development of Functional Connectivity

**Figure 4:**
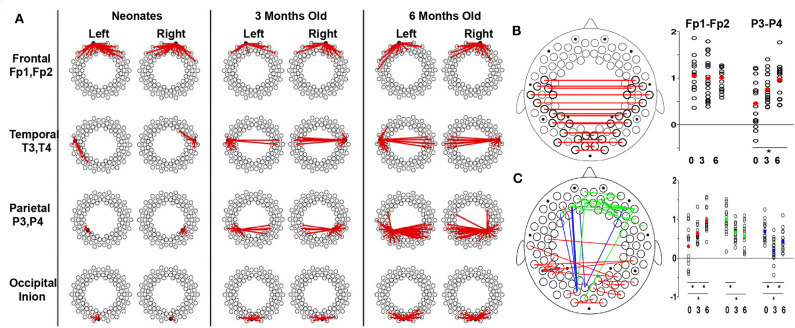
Developmental trajectories of functional connectivity. **(A)** Development of the connectome with seeds in different brain regions. The dots represent the measurement channels, and the red lines represent correlations higher than 0.5. **(B)** Development of the connectome in homologous channels. Red lines represent the connections exhibiting significant changes. The scatter plots show *z* scores in frontal (Fp1–Fp2) and parietal (P3–P4) pairs, with the red dot indicating the average values. **(C)** Increase (red), decrease (green), and *U*-shaped (blue) changes in the connectome across development. The normalized connectivity grouped by the change patterns are displayed next to the brain template, in which the dots with corresponding colors represent the average connectivity of corresponding change patterns. For **(B,C)**, the significant differences are labeled with asterisks (*p* < 0.05, *post-hoc* tests, Tukey's HSD). These figures are adapted from Homae et al. (2010) under a Creative Commons Attribution-Non-commercial-Share Alike 3.0 Unported License (CC BY-NC-SA 3.0, https://creativecommons.org/licenses/by-nc-sa/3.0/).

Functional connectivity is a potential biomarker of brain development and has been frequently used in the fNIRS community recently. For example, Ferradal et al. (2016) used a high-density diffuse optical tomography imaging system to record the spontaneous brain activity in healthy, full-term neonates within the first 2 days of their life. Seed-based correlation analysis was used to identify intrinsic functional connectivity in infants. In their study, strong connectivity was found between homotopic counterparts of each region of interest (e.g., visual, middle temporal visual area, and auditory cortices), indicating a well-developed functional connectivity in infants. These results also highlight the fNIRS imaging system as a powerful and practical method for quantitative mapping of the early functional brain development in neonates. With the development of the brain, functional connectivity undergoes substantial changes, which demonstrate the strengthening and pruning of brain connectivity between different cortical regions (Damaraju et al., 2014; Cao et al., 2017b). For example, during the first 6 months of life, Homae et al. (2010) presented that the cortical network organization showed region-dependent and dynamic changes (Figures 4A–C). Specifically, the functional connectivity between homologs regions was identified in three- and four-month-old infants (Homae et al., 2010, 2011; Funane et al., 2014; Blanco et al., 2018). Homae et al. (2010) further identified the age-related enhanced homolog functional connectivity in the bilateral temporal, parietal, and occipital cortices (Figures 4A,B) and in the temporal and posterior regions (Figure 4C). This implies the strengthening and pruning of connections between different cortical regions during the development of the infant brain (Figure 4). Notably, an “*U*-shaped” developmental pattern for edges connecting frontal and occipital regions during development was also observed. For example, frontoposterior long-connectivity interactions decreased from the neonatal period to the age of 3 months and increased from the age of 3 months to the age of 6 months (Figure 4C; Homae et al., 2010). By contrast, the functional connectivity within the temporal regions, which was enhanced from 0 to 6 months in Homae et al.'s study, exhibited obviously decreased in 4- to 10-months-old infants compared to that in infants aged 2 to 3 months old (Taga et al., 2018).

The dynamic changes in brain network may benefit the cognition and behavior. As was pointed out by Li and Qiu et al., 9-years-old children exhibited significantly decreased functional connectivity in language areas when compared to adults, which demonstrates that functional connectivity in children remains immature (Li and Qiu, 2014). By examining 18-months-old toddlers, Bulgarelli et al. (2019) showed that infants who developed good self-awareness exhibited increased functional connectivity between frontal and temporoparietal regions. Note that such connectivity between frontal region and posterior region has been identified in infants aged as early as 4 months old (Blanco et al., 2018).

### Development of Brain Network Topologies

**Figure 5:**
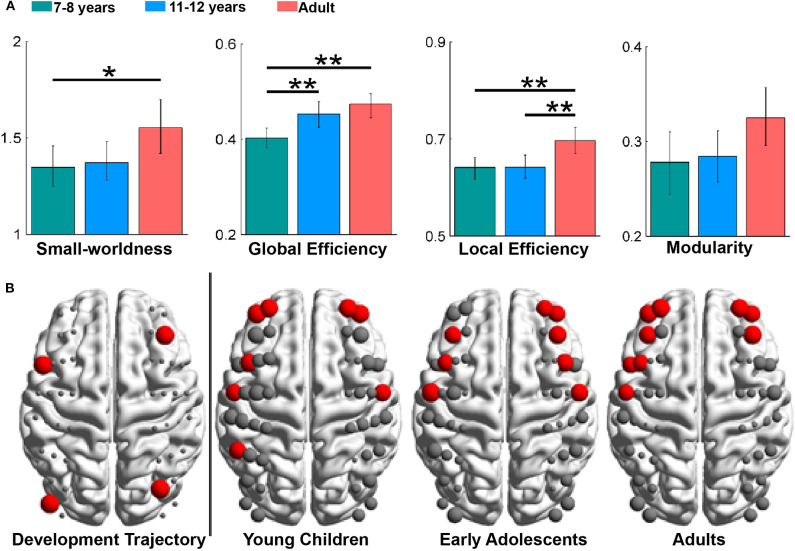
Developmental trajectories of brain network topologies and asymmetry. **(A)** Group differences in the global network metrics, with significance indicated by asterisks (*p < 0.05, **p < 0.01). **(B)** The developmental trajectory (left) and hub distributions (right). In the developmental trajectory, nodes exhibiting age-related increases in nodal efficiency are shown in red. In the hub distributions, hubs are also shown in red, and the node size represents the values in the nodal properties. These figures are adapted from Cai et al. (2018) under an Attribution-Non-Commercial-NoDerivatives 4.0 International License (CC BY-NC-ND 4.0, https://creativecommons.org/licenses/by-nc-nd/4.0/).

Global and local network properties are often used to characterize integration and segregation of the developing brain. For example, Cai et al. (2018) found that global and local network efficiency in children and adolescents was significantly lower than that in adults (Figure 5A), demonstrating immature global information processing and local information transformation in children and adolescents. Intriguingly, children showed a significantly lower global efficiency, but comparable local efficiency, compared to early adolescents, which indicates enhance integration and steady segregation of the functional network from childhood to adolescence. Furthermore, it was found that the small-worldness and modularity showed no significant differences across developmental stages (Figure 5A), demonstrating maturity of these important functional brain network organization properties in the early stage of childhood.

For the regional nodal metrics, it was found that nodes with a significant increase in nodal efficiency during development were mainly distributed in frontal brain regions (Figure 5B), and the number of frontal hubs increased with development. Consistent with previous studies showing late developed and enhanced functional connectivity in the frontal regions (Gao et al., 2015a,b), this result provides additional biomarkers for the increasing cognitive capacity during the development.

### Development of Brain Network Asymmetry

Cerebral asymmetry is a fundamental characteristic of the human brain and an important marker of brain development. Gallagher et al. (2016) compared resting-state functional connectivity identified in language regions to the activation maps of language tasks in 25 children. They found good agreement between these two approaches for language localization and hemispheric language dominance (Figure 3A). These results provide preliminary evidence that the fNIRS-derived functional connectivity is a valuable tool for language localization.

**Figure 6:**
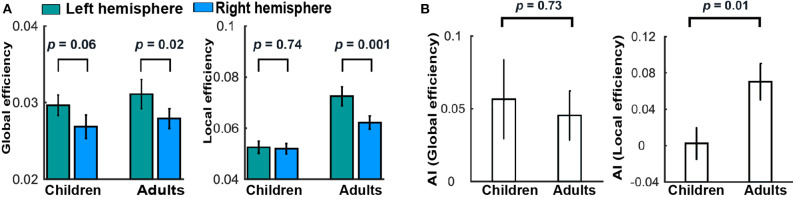
Development of brain asymmetry. **(A)** Differences between the hemispheres in global and local efficiency for each group. **(B)** Group differences in the AI of global and local efficiency. These figures were adapted from Cai et al. (2018) under a CC BY-NC-ND 4.0 license.

In addition, Cai et al. (2019) investigated the asymmetry of the brain network properties and their development from childhood to adulthood. They found the leftward asymmetry in global but not local efficiency in children (Figure 6A). For the adults, both hemispheric global and local efficiency showed a significant leftward asymmetry (Figure 6A). It was further revealed by asymmetry index (AI) that the leftward asymmetry in local (but not global) network efficiency significantly increased with development (Figure 6B). At the regional level, increased leftward asymmetry in nodal efficiency with development was mainly observed in the frontal, parietal–occipital junction, and occipital regions. Collectively, these developmental patterns of topological asymmetries suggest that the maturation of functional segregation in the left hemisphere plays a more important role than the right hemisphere in the cognitive development from childhood to adulthood.

## Atypical Development of the Brain Connectome

In this part, we introduce the findings regarding abnormal brain networks in preterm infants and children with neural developmental disorders (e.g., cerebral palsy, Down's syndrome, and autism spectrum disorder) using the resting-state fNIRS brain connectome.

### Brain Connectome in Preterm Infants

**Figure 7:**
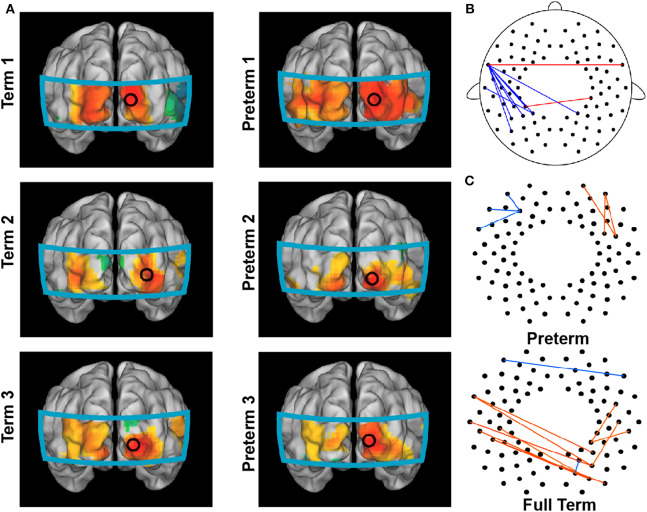
Brain connectome in preterm infants. **(A)** Functional connectivity maps in full-term infants (left column) and preterm infants (right column) with the seed located in the right occipital region. **(B)** Group differences between preterm and full-term infants in functional connectivity. Red lines indicate the enhanced connections of preterm infants, and blue lines indicate the decreased connections of preterm infants compared to those of full-term infants. **(C)** Connections exhibiting significant correlations between normalized functional connectivity and postmenstrual age at the time of the scan in preterm (up) and full-term (bottom) infants. **(A)** is adapted from White et al. (2012) with permission from Elsevier; **(B,C)** are adapted from Fuchino et al. (2013) under a CC BY 4.0 license.

In 2012, White et al. showed that functional connectivity could be obtained at the bedside using fNIRS imaging. They identified functional connectivity in the visual cortex in both healthy and preterm infants (Figure 7A). Interestingly, Fuchino et al. (2013) noted that preterm infants showed enhanced connectivity between bilateral temporal regions and parietal regions compared to that in postmenstrual age-matched full-term infants (Figure 7B). However, preterm infants' functional connectivity between the left temporal and left parietal regions was much lower than full-term infants (Figure 7B). These findings imply that preterm infants have developed sufficient connectivity between homologous language-related regions (i.e., temporal cortex). However, their language development was still influenced by the immature connectivity between the temporal cortex and posterior regions, which should have increased with age (Figure 4C, Homae et al., 2010). This interpretation was further confirmed by the developmental trajectories of preterm and full-term infants (Figure 7C), in which the age-related development mainly occurred in frontal areas in the preterm infants, while changes in connectivity were mainly identified between the left temporal and posterior regions in the full-term infants (Fuchino et al., 2013).

### Brain Connectome in Children With Neurological Disorders

**Figure 8:**
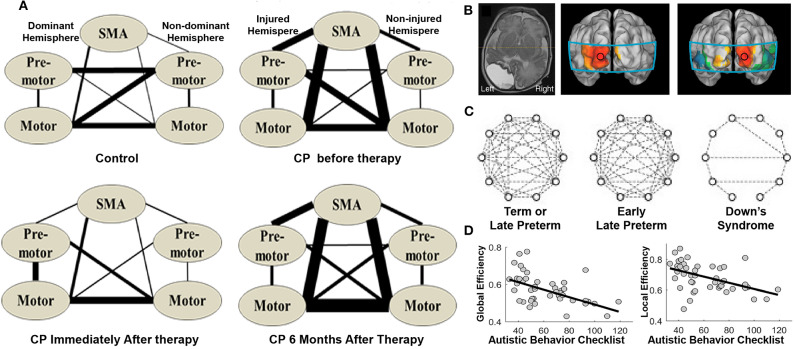
Brain connectome in children with neurological disorders. **(A)** Brain connectome changes in cerebral palsy patients (CP) before, immediately after, and 6 months after the therapy. The thickness of the lines indicates the percentage of subjects with significant connectivity. **(B)** Structural MRI of a preterm infant with stroke and its functional connectivity maps with seeds in the left and right occipital cortex, respectively. **(C)** Brain connectome in each group. The dashed lines indicate correlation coefficients higher than 0.5. **(D)** Scatter plot illustrating the relationship between autistic behaviors and network efficiency (i.e., global and local efficiency). **(A)** is adapted from Cao et al. (2015) under a CC BY 4.0 license; **(B,C)** is adapted from White et al. (2012) and Imai et al. (2014), respectively, with permission from Elsevier; **(D)** is adapted from Li and Yu (2018) under a CC BY 4.0 license.

It is well-established that disruptions in functional connectivity and topological network organization underlie various neurological disorders (Suo et al., 2015; Li et al., 2018; Wang et al., 2020). Meanwhile, resting-state fNIRS has been widely used to capture network dysfunctions in the early stage and to aid diagnosis of many pediatric neurological conditions. For example, 10-years-old children with cerebral palsy exhibited increased functional connectivity between sensorimotor centers, which was back to normal immediately after physical therapy but then relapsed after 6 months (Figure 8A; Cao et al., 2015). White et al. (2012) examined the functional connectivity pattern in an infant with unilateral occipital stroke and found that the functional connectivity in the visual cortex was unilateral and nonsymmetrical (Figure 8B). For healthy infants, the connectivity displayed a strong and bilaterally symmetrical pattern in the occipital network. In addition, for infants with Down's syndrome, the authors found decreased functional connectivity in the frontal, temporal, and occipital regions compared to that in full-term and preterm infants (Figure 8C; Imai et al., 2014). Decreased functional connectivity was also observed in the bilateral temporal cortices of children with autism spectrum disorder (ASD) compared to that in typically developing children (Zhu et al., 2014; Li et al., 2016). Furthermore, children's autistic behaviors were associated with decreased network efficiency (Figure 8D; Li and Yu, 2018). These findings support that resting-state networks, as characterized by resting-state fNIRS, are sensitive to neural dysfunctions. Therefore, disruptions in the fNIRS-based brain connectome could serve as a biomarker in diagnosis and in the evaluation of rehabilitation.

## Concluding Remarks and Further Considerations

The current review clearly demonstrates the validity of the fNIRS-based brain connectome. By using this technique, a lot of studies have illustrated the typical and atypical development in resting-state functional connectivity and topological organization of brain networks from the neonatal period to adolescence. More specifically, functional brain connectivity was identified in neonates (Ferradal et al., 2016), and it changed dynamically with development, which reflects the strengthening and pruning of brain connectivity during development. Several topological properties of the functional networks in children were similar to those of adults, e.g., in small-world and modularity characteristics, which indicated the developmental maturity in the network organization in childhood. However, it should be noted that some specific network properties, e.g., global efficiency and local efficiency, are still being optimized during the development (i.e., both increased segregation and integration; Cai et al., 2018). Interestingly, from the view of hemispheric lateralization, local efficiency also showed a significant increase in the topological left asymmetry with development from childhood to adulthood (Cai et al., 2019). These accumulated evidence highlights that the maturation of functional segregation in the left hemisphere plays a more important role than the right hemisphere in the cognitive development from childhood to adulthood. Furthermore, resting-state fNIRS can also capture network dysfunctions in the atypically developing brain, which often leads to different neurological disorders such as autism (Li and Yu, 2018), Down's syndrome (Imai et al., 2014), and cerebral palsy (Cao et al., 2015). The abovementioned findings illustrate that fNIRS-derived connectome can serve as a promising tool for mapping neural development and as a biomarker for neurological diagnosis and rehabilitation evaluation.

However, several issues still need to be addressed in the future. First, these findings were derived from cross-sectional data, which could be influenced by inter-subject variability and unmatched cohort distributions. No longitudinal studies regarding the fNIRS connectome development have been conducted, which should be conducted in the future to reveal the nature of developmental changes.

Second, topological development has not been fully explored by these studies, and other network properties, such as network variability (Li et al., 2015), betweenness centrality (Zhao et al., 2018), and causality (Hu et al., 2019), await further investigation. Additionally, more methodological work should be performed to validate these analytical approaches.

Third, we have not combined the fNIRS with other imaging modalities such as electroencephalograph (EEG) to achieve a more comprehensive understanding of the developing brain network. The simultaneous EEG-fNIRS recording has been used in clinical monitoring (Kassab et al., 2018) and addiction research (Ieong and Yuan, 2019). It should be used in the investigation of the developing brain to depict the developmental trajectories of neurovascular coupling.

Fourth, most developmental studies did not investigate the relationship between brain connectome metrics (e.g., functional connectivity, cerebral asymmetry, and network characteristics) and behavioral performance. It remains largely unknown regarding to the underlying physiological basis of behavior performances at different stages of brain development. Further studies employing multimodal imaging and longitudinal data should be conducted to ascertain the brain-behavior relationship during development.

Fifth, the infant participants included in these studies were always in a sleeping state, while children in other age stages were in an awake state. Since, Taga et al. (2018) found that stimuli during wakefulness and sleep elicited different cortical response, the states during the resting-recording may also influence the resting-state networks, which also deserve further investigation.

Sixth, resting-state fNIRS imaging should be included in routine introspection in clinical psychiatric practice. Because of the convenient operating procedure, fNIRS is among the best candidates for clinical use. The quickly accumulating neurological data from this technique combined with the emerging deep learning technology (Hennrich et al., 2015; Marblestone et al., 2016; Cole et al., 2017; Shen et al., 2017; Vieira et al., 2017; Zhou et al., 2018) could help to diagnose neurological diseases precisely.

## Conclusion

This review summarizes current advances in resting-sate fNIRS in characterizing the development of the brain connectome. By illustrating the brain connectome structure and quantifying the topological properties, dozens of fNIRS studies have suggested that fNIRS is an ideal alternative to resting-state fMRI for demonstrating the developmental trajectories of spontaneous cortical connectome. Despite the fruitful achievements in this field, we suggest that future studies pay more attention to the longitudinal data, expand the methodological approaches and measure modalities, ascertain the brain-behavior relationship, compare the brain states, increase the sample size, and make the best use of deep learning technology. As such, resting-state fNIRS could pave a new way toward a better understanding of brain network development.

## Author Contributions

ZH, QD, and HN designed the framework of this review. ZH and GL conducted the search, selected the relevant literature, and made the tables and figures. ZH wrote the manuscript. All authors reviewed the manuscript and made significant contributions to this manuscript.

## Conflict of Interest

The authors declare that the research was conducted in the absence of any commercial or financial relationships that could be construed as a potential conflict of interest.
